# Capreomycin is active against non-replicating *M. tuberculosis*

**DOI:** 10.1186/1476-0711-4-6

**Published:** 2005-04-01

**Authors:** Leonid Heifets, Julie Simon, Van Pham

**Affiliations:** 1 National Jewish Medical and Research Center, Denver, Colorado 80206, USA; 2 6955 S.W. Larkspur Pl., Beaverton, OR 97008, USA

**Keywords:** latent tuberculosis infection, dormant tubercle bacilli, capreomycin

## Abstract

**Background:**

Latent tuberculosis infection (LTBI) is affecting one-third of the world population, and activation of LTBI is a substantial source of new cases of tuberculosis. LTBI is caused by tubercle bacilli in a state of non-replicating persistence (NRP), and the goal of this study was to evaluate the activity *in vitro *of various antimicrobial agents against non-replicating *M. tuberculosis*.

**Methods:**

To achieve a state of NRP we placed broth cultures of *M. tuberculosis *(three strains) in anaerobic conditions, and in this model tested all known anti-TB drugs and some other antimicrobial agents (a total of 32 drugs). The potential effect was evaluated by plating samples from broth cultures for determining the number of viable bacteria (CFU/ml) during a prolonged period of cultivation. Besides drug-free controls we used metronidazole for positive controls, the only drug known so far to be effective against tubercle bacilli in anaerobic setting.

**Results:**

On a background of non-replicating conditions in drug-free cultures and clear bactericidal effect of metronidazole none of the antimicrobial agents tested produced effect similar to that of metronidazole except capreomycin, which was as bactericidal at the same level as metronidazole.

**Conclusion:**

The unique ability of capreomycin to be bactericidal *in vitro *among the anti-TB drugs against non-replicating tubercle bacilli may justify the search for other drugs among peptide antibiotics with similar activity. This phenomenon requires further studies on the mechanism of action of capreomycin, and evaluation of its activity in appropriate animal models.

## Background

Latent tuberculosis infection (LTBI) is defined as a clinical condition without clinical or radiological signs of active disease, and it is manifested only by a positive tuberculin skin test [1]. Approximately two billion people, or one-third of the world's population, have LTBI, and approximately 10% of them will develop active TB during their lifetime. It is also estimated that one-third of all new cases of active TB (about 2.5 million cases reported annually in the world), results from activation of LTBI. The role of LTBI is even greater in persons co-infected with HIV. Currently, 11 million people in this category are registered throughout the world, and at least 10% of them develop active TB every year. All these facts have placed the problem of diagnosis and effective treatment of LTBI in the frontier of current TB research agenda.

There is plenty of evidence that the basis for LTBI in humans is persistence of tubercle bacilli *in vivo *for long periods of time. This status is currently defined as dormancy or non-replicating persistence (NRP) emphasizing that each of these terms is only descriptive, regardless of the mechanisms involved [2]. An important element of this definition is that the suggested terms indicate not only the constant number of tubercle bacilli over time (stationary phase of growth *in vitro*), but actual lack of multiplication. Besides LTBI, a part of bacterial population in patients with active disease also persists in a non-replicating state, and there is an obvious need for a drug affecting this sub-population – a drug that would be considered for incorporation into treatment regimens.

The *in vivo *factors that may induce NRP include depletion of nutrients, shifts in pH, accumulation of growth inhibiting products, and depletion of oxygen. Discovery of effective drugs against non-replicating tubercle bacilli requires appropriate *in vitro *models of dormancy. In the past, the following *in vitro *models were suggested: cultivation at 8°C for 31 days [3], nutrient depletion [4], depletion of oxygen in vigorously shaken broth cultures [5], anaerobic model in submerged broth cultures [6], and cultivation at pH 4.8–5.0 in broth for six weeks [7]. Not all of these models fully comply with the above definition of dormancy, and the most popular among them became the anaerobic model by Wayne [6]. The physiological parameters of NRP of *M. tuberculosis *in this model were studied intensively [8-12], which makes the anaerobic conditions the most attractive tool for further studies of NRP.

The aim of this study was to analyze the activity of all available anti-tuberculosis drugs in broth cultures in *in vitro *anaerobic conditions. Only capreomycin was found to be bactericidal in these conditions, and the activity of this drug was at the same level as that of metronidazole.

## Methods

### Antimicrobial agents

Metronidazole was obtained from Searle (Skokie, IL). All other drugs and reagents were obtained from Sigma (St. Louis, MO). Stock solutions of rifampin were made in methanol, ethionamide – in DMSO, and all other drugs – in distilled water. Working solutions were prepared from stock solution in 7H9 broth.

### Test-strains

Three drug-susceptible strains of *M. tuberculosis*, H_37_Rv, Erdmann, and Atencio, were kept in aliquots of 7H9 broth culture frozen at -70°C. The inocula were prepared by 5–7 days of sub-cultivation in fresh 7H9 broth tubes (4.5 ml) placed in a roller drum for better aeration. These cultures were used as inocula when the turbidity became equal to the optical McFarland standard #1, which corresponded to about 10^8 ^CFU/ml.

### Inoculum for hypoxia *in vitro *model

The so-called "anaerobic inoculum" of tubercle bacilli was prepared according to the original description [6,13]. According to these reports, with conditions of gradually decreased supply of oxygen, the tubercle bacilli undergo an orderly metabolic down-shift into a state of dormancy when the bacteria accumulate in the bottom of the unshaken tubes (20 × 125 mm) containing 10 ml of broth. In our experiments, a bacterial suspension (optical density at #1 McFarland standard) was made from 10–14 day-old 7H11 agar culture. After homogenization with glass beads, the suspension was placed into 25 × 125 mm tubes, 4 ml in each, and an additional 6.0 ml of fresh 7H9 broth was added. After cultivation at 37°C for 19 days in an undisturbed upright position, the caps were loosened by 1/2 turn, and the tubes were placed into an anaerobic jar for another nine days of cultivation. The jars contained indicator strips and GasPackPlus envelopes filled with 10 ml of sterile water. Sediments were removed from tubes by inserting pipettes through supernatants, and then combined into one tube, approximately 8 ml from each four tubes set.

### Medium for anaerobic cultivation

Standard 7H12 broth in 12B Bactec vials (4.0 ml) was supplemented with three aqueous filter-sterilized reagent solutions, each added in a volume of 0.1 ml per vial. Two of them were oxygen-reducing reagents: Sodium thioglycolate and L-cysteine hydrochlorate. The third was methylene blue, a color indicator of oxygen presence. The final concentration was 0.5 mg/ml of each of these reagents in the medium.

### Hypoxia *in vitro *model: experiments with drugs

We used the Bactec-460 system set in an anaerobic mode for testing activity of drugs in anaerobic conditions. After addition of the above-described reagents, the bottom edges of the caps of the12B vials were sealed by coating with rubber cement, the drug solutions were added (0.1 ml per 4.0-ml vial), and the vials were run through the Bactec machine with an anaerobic setting using Nitrogen gas + 5% CO_2_. The vials were inoculated with the described above bacterial suspension (0.1 ml per vial). The vials were subsequently incubated at 37°C, and checked daily for a possible change in color. Those with blue color (disruption of anaerobic conditions) were discarded. The vials were run through the anaerobic Bactec-460 system every four days to record the cumulative Growth Index (GI). To determine the number of viable bacteria in these cultures, we took samples from alternate vials, starting at day zero, and subsequently every week, for plating appropriate dilutions on 7H11 agar plates. The results were expressed in CFU/ml at each time-point.

### Statistical analyses

We have determined the regression coefficients for each of the curves of decline in bacterial counts (CFU/ml) in the presence of all drugs, as well as in the drug-free controls. For each coefficient we determined confidence limits for the 0.95 probability, as well as its level of statistical significance (difference from the "no-decline") expressed in the *p *value. In addition, we determined the statistical difference between the regression coefficients for each drug, on one hand, and the same values found for drug-free controls, also expressed as a *p *value.

## Results

### Model validation

**Figure 1:**
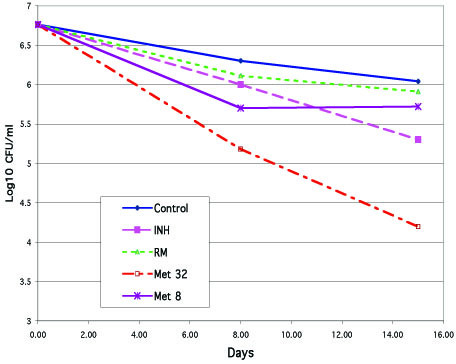
One of experiments confirming the validity of the anaerobic model: kinetics of CFU/ml of *M. tuberculosis *on days 0, 8, and 15 in 7H12 broth in anaerobic conditions: in drug-free control, in the presence of metronidazole (Met, 32 and 8 μg/ml), rifampin (RMP, 0.5 μg/ml), and isoniazid (INH, 0.5 μg/ml).

We evaluated the kinetics of GI and CFU/ml in three preliminary experiments (with three strains) using vials without any drugs added and in the presence of metronidazole. Cultivation in these preliminary experiments continued up to 29 days, and samples for CFU/ml determination were taken from alternate vials on days 0, 4, 8, 11, 15, 18, 22, 25, and 29. During this period of time, production of CO_2 _in cultures remained at a steady low level (with daily GIs less than 10), when cumulative four-day readings on the Bactec-460 machine did not exceed GI = 40. The number of CFU/ml in drug-free vials remained relatively steady during this period, with only a small decline within the first 18 days. The kinetics of CFU/ml in vials containing metranidazole was different: a decline could have been detected at day 8, but it became more evident (by one-to-two log_10_) at day 15. This decline in the number of CFU/ml was more significant in the presence of 32 μg/ml of metronidazole than at 8.0 μg/ml. This difference compared to the drug-free medium and between the effects of two drug concentrations became greater during the subsequent period. Nevertheless, we found these differences within the first 15 days of cultivation quite sufficient and less laborious for the subsequent 15 validation experiments (five with each of the test-strains), in which the number of CFU/ml was determined on days 0, 8, and 15. In addition, we incorporated two more agents along with metronidazole (rifampin 0.5 μg/ml and isoniazid 0.5 μg/ml) into these experiments. The summary of these experiments, presented in Table 1, demonstrated results similar to that in the original report regarding activity of these agents in anaerobic conditions (13): clear dose-dependent bactericidal effect of metronidazole, very slight effect of rifampin, and no effect of isoniazid. The results of one of these experiments is also shown in Fig. 1.

**Table 1:** Antimicrobial Effect of Metronidazole (Met), Rifampin (RMP) and Isoniazid (INH)Against Three Strains of *M. tuberculosis*

Drug/ Conc. (μg/ml)	Log_10 _of CFU/ml on days 0, 8 and 15								
	
	H_37_Rv			Atencio			Erdman		
	
	0	8	15	0	8	15	0	8	15
Control	6.76	6.30	6.04	7.26	7.49	7.34	7.08	6.63	6.30
Met. 32.0	6.76	5.18	4.20	7.26	5.73	5.76	7.08	4.70	4.18
Met. 8.0	6.76	5.70	5.72	7.26	6.49	6.20	7.08	5.88	5.18
RMP 0.5	6.76	6.11	5.91	7.26	6.50	6.50	7.08	5.76	5.59
INH 0.5	6.76	6.00	5.30	7.26	7.00	7.00	7.08	6.00	5.87

### Screening of other drugs

In subsequent experiments, each drug was tested in three concentrations (μg/ml): amikacin (8, 4, 2), capreomycin (8, 4, 2), ciprofloxacin (4, 2, 1), clarithromycin (32, 8, 2), ethambutol (8, 4, 2), ethionamide (4, 2, 1), gatifloxacin (2, 0.5, 0.12), isoniazid (2.5, 0.5, 0.1), levofloxacin (2, 1, 0.5), PAS (25 and 6.25), pyrazinamide at pH 6.0 (900, 300, 100), morphazinamide (300 and 100), rifabutin (0.5, 0.25, 0.12), rifampin (0.5, 0.25, 0.12), rifapentine (0.12, 0.06, 0.03), streptomycin (8, 4, 2), sparfloxacin (2, 1, 0.5), thiacetazone (1.2, 0.3, 0.075). Other drugs were tested in single concentrations: amoxicillin, augmentin, clindomycin, ceftriaxone, dapsone, doxycycline, erythromycin, cefoxitin, gentamycin, imipenem, minocycline, pristomycin, sulfamethoxazole, tetracycline, trimethoprim. Each experiment for testing two or three of the above listed drugs always included a drug-free control and metronidazole. The analyses included only those experiments in which clear difference in the kinetics of CFU/ml between drug-free controls and metronidazole have been observed.

**Figure 2:**
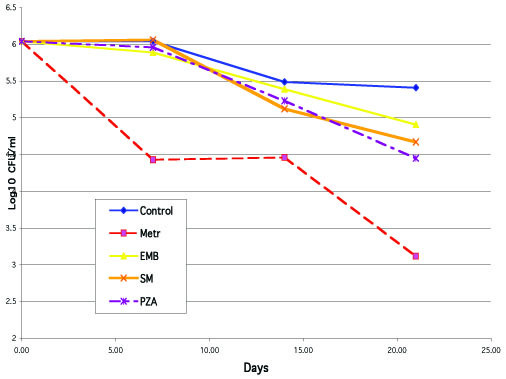
Kinetics of CFU/ml of *M. tuberculosis *in anaerobic conditions on days 0, 7, 14, and 21 in one of experiments with conventional anti-TB drugs compared to that of the drug-free control and effect of metronidazole (Met. 16 μg/ml): ethambutol (EMB, 8 μg/ml), streptomycin (SM, 8 μg/ml), and pyrazinamide (PZA, 900 μg/ml, at pH 6.0).

Other criteria for inclusion were: no appearance of blue color in the media, and no greater than GI = 50 cumulative readings at days 7–8, 14–15, and 21. These screening experiments clearly demonstrated that none of the above listed agents, except capreomycin (see below), could produce any antimicrobial activity in the model used, contrasting with clear bactericidal activity of metronidazole. The results of some of these experiments are shown in Table 2 and Fig. 2.

**Table 2:** Kinetics of the Number of Viable Bacteria (Log_10 _CFU/ml). Initial CFU/ml Contents: H37Rv – 6.32, Atencio – 6.75, Erdman – 6.52.

Drug/ Conc. (μg/ml)	Log_10 _of CFU/ml on days 7, 14 and 21								
	
	H_37_Rv			Atencio			Erdman		
	
	7	14	21	7	14	21	7	14	21
Control	6.04	5.49	4.91	5.30	5.62	4.86	5.90	5.32	4.91
MET, 16	4.43	4.46	3.12	4.54	3.84	3.15	4.04	3.67	3.28
EMB, 8	5.86	5.39	4.91	6.17	5.30	4.71	5.82	5.28	4.72
SM, 8	6.06	5.12	4.67	6.07	5.20	4.31	5.75	5.16	4.46
PZA, 900*	5.96	5.23	4.45	5.96	5.08	4.16	5.59	5.17	4.50

*At pH 6.2

### Activity of capreomycin in anaerobic cultures

**Figure 3:**
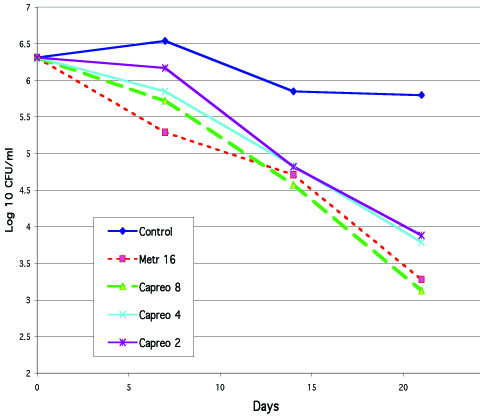
Effect of capreomycin (CM, at 8, 4, and 2 μg/ml) in one of experiments against *M. tuberculosis *(H37Rv) in anaerobic conditions compared to that of metronidazole (Met, 16 μg/ml)

A series of 29 experiments (six of which included capreomycin) were conducted with assessment of the number of CFU/ml at four time-points: days 0, 7, 14, and 21. Some decline in the number of viable bacteria, at one log_10 _or less, occurred in drug-free controls during this period. Metronidazole used in a concentration of 16 μg/ml in these experiments produced a more significant effect, with a difference of 1–2 log_10 _or more compared with the number of CFU/ml in drug-free controls at day 21 of cultivation (Table 3). The effect of capreomycin at a concentration of 8.0 μg/ml in these experiments was similar to that of metronidazole, and clear dose-response relations was seen for this effect (Table 3 and Fig. 3).

**Table 3:** Antimicrobial Effect of Capreomycin (CM) at Concentrations of 8, 4, and 2 μg/ml Initial CFU/ml Content: H37Rv – 7.28, Atencio – 6.31, Erdman – 6.88

Drug. Conc.	Log_10 _of CFU/ml on days 7, 14 and 21								
	
	H_37_Rv			Atencio			Erdman		
	
	7	14	21	7	14	21	7	14	21
Control	6.54	5.85	5.80	6.25	6.30	4.92	6.78	5.20	5.31
MET, 16	5.29	4.71	3.28	5.37	6.54	3.82	6.08	5.00	3.88
CM, 8	5.72	4.57	3.13	5.56	5.97	3.24	5.68	4.88	3.18
CM, 4	5.85	4.83	3.79	5.60	6.13	3.36	5.65	5.02	3.93
CM, 2	6.17	4.82	3.88	5.83	6.26		5.62		4.71

### Statistical significance

Decline in the number of viable bacteria in drug-free controls was not significant, with the exception being for the strain Erdman in experiments presented in Table 1 and 2 (*p *< 0.05). Decline in the number of viable bacteria in the presence of RMP and INH (Table 1) and EMB and SM (Table 2) was not significant in experiments with strains H37Rv and Atencio (*p *> 0.05). Decline in CFU/ml in the presence of metranidazole 32.0 and 16.0 μg/ml was statistically significant (*p *< 0.05) in all experiments, with best results in experiments with the H37Rv strain, showing most significant difference from decline in controls. Capreomycin (Table 3) was most active against strain H37Rv at concentrations of 8.0 and 4.0 μg/ml (*p *= 0.002 and *p *= 0.004, correspondingly) with definitive difference from the curve of decline in the control (*p *= 0.003 and *p *= 0.009, correspondingly).

## Discussion

We examined the antimicrobial activity of all anti-TB drugs and of other antimicrobial agents against *M. tuberculosis *in anaerobic conditions. For this purpose, we used an anaerobic *in vitro *model based on the same principles suggested by Wayne for submerged broth culture cultivation (6,13). Preparation of the "anaerobic inoculum" of *M. tuberculosis *was identical to the procedure described in these reports. On the other hand, to evaluate the activity of various drugs, we used a different technology (the Bactec-460 system) for subsequent cultivation in anaerobic conditions. One of the advantages of this system is that it is less laborious for testing a large number of drugs than the original technique. Running the culture vials through the Bactec-460 anaerobic system with a nitrogen gas flow effectively provided anaerobic conditions, enforced by oxygen-reducing agents and controlled by the color indicator present in each vial. In the past, it was established that daily release of CO_2_, which was automatically recorded in this system as radiometric Growth Index (daily GI), correlates well with the increase in the number of viable bacteria. Therefore, detection of any significant amount of CO_2 _released in cultures in any of the vials provided an instant indication of possible growth, and such vials could have been immediately removed from the experiment. The validity of this system was confirmed in a series of experiments to test the activity of metronidazole, rifampin, and isoniazid. Results obtained in these experiments were identical to those reported by Wayne and Sramek [13].

Among all drugs listed in the Methods section, only capreomycin exhibited a clear bactericidal effect similar to that of metronidazole against non-replicating *M. tuberculosis *in anaerobic conditions, and this finding was confirmed in six experiments with three *M. tuberculosis *strains.

We previously reported that the MICs of capreomycin in aerobic conditions against actively multiplying *M. tuberculosis *were within the range of 1.25–2.5 μg/ml in either liquid or solid (7H11 agar) medium, and the MBC/MIC ratio was equal to 2, similar to that of streptomycin, amikacin, and kanamycin [14,15]. New findings that capreomycin can be active against non-replicating *M. tuberculosis *require further investigation, particularly in appropriate animal models. The unusual phenomenon of such activity also requires further studies in regard to the mode of action of this drug in anaerobic conditions. It is well known that *M. tuberculosis *in NRP state represent a problem of LTBI, but also can be a part of the bacterial population in new patients with active tuberculosis [16]. Therefore, it is quite possible that capreomycin may not only be effective in patients with LTBI, but also may play an important role as an element of an initial treatment regimen in new patients with active tuberculosis. Either of these possibilities should be investigated in various animal models and subsequently considered for confirmation in clinical trials. Setting up and conducting a large-scale trial with LTBI patients would require a substantial period of observation and can be quite expensive. Therefore, before addressing the issue of treatment of LTBI patients, another option that can be considered is evaluation of the potential effect of capreomycin (in a combination with other drugs) for newly diagnosed patients. This can be achieved in pilot clinical trials using various surrogate markers to determine bactericidal and sterilizing activity of drugs or drug combination [17-19]. It is premature to define the exact avenues of clinical studies until the needed information on the effect of capreomycin in appropriate animal models becomes available.

## Conclusion

Among the known anti-TB drugs and other tested agents, only capreomycin exhibited a bactericidal effect against non-replicating *M. tuberculosis *in anaerobic conditions *in vitro*. Further studies are needed to investigate the mode of action of this drug in such a setting, as well as the efficacy in appropriate animal models, and, perhaps, in pilot clinical trials. Testing in an anaerobic dormancy model of some other polypeptides may lead to the discovery of other drugs effective against non-replicating *M. tuberculosis*.

## Authors' contributions

LH directed the study, analyzed and interpreted the results, drafted the manuscript. JC contributed to the design of the model, and performed experiments with anti-TB drugs. VP contributed to the standardization of the procedures, especially for quality controls of anaerobic conditions, standardized CFU/ml quantitation, repeated a series of experiments with anti-TB drugs and conducted experiment with other antimicrobials.
